# Microscale mobile surface double layer in a glassy polymer

**DOI:** 10.1126/sciadv.abq5295

**Published:** 2022-11-09

**Authors:** Hailin Yuan, Jinsong Yan, Ping Gao, Sanat K. Kumar, Ophelia K. C. Tsui

**Affiliations:** ^1^ Department of Physics, Hong Kong University of Science and Technology, Clear Water Bay, Hong Kong, China.; ^2^ William Mong Institute of Nano Science and Technology, Hong Kong University of Science and Technology, Clear Water Bay, Hong Kong, China.; ^3^ Department of Chemical and Biological Engineering, Hong Kong University of Science and Technology, Clear Water Bay, Hong Kong, China.; ^4^ Department of Chemical Engineering, Columbia University, New York, NY 10027, USA.

## Abstract

This study examines the origin of the widely different length scales, *h*_t_—nanometers to micrometers—that have been observed for the propagation of the near-surface enhanced mobility in glassy polymers. Mechanical relaxations of polystyrene films with thicknesses, *h*, from 5 nm to 186 μm have been studied. For *h* < ~1 μm, the films relaxed faster than the bulk and the relaxation time decreased with decreasing *h* below ~100 nm, consistent with the enhanced dynamics originating from a near-surface nanolayer. For *h* > ~1 μm, a bulk-like relaxation mode emerged, while the fast mode changed to one that extended over ~1 μm from the free surface. These findings evidence that the mobile surface region is inhomogeneous, comprising a nanoscale outer layer and a slower microscale sublayer that relax by different mechanisms. Consequently, measurements probing the enhanced mobility of different mechanisms may find vastly different *h*_t_’s as shown by the literature.

## Teaser

The mobility of polymer glass is enhanced within micrometers of the surface but is further enhanced within the outer nanometers.

## INTRODUCTION

The glass transition occurs when the molecular motions of a substance slow down markedly on cooling. This is the process by which amorphous polymers transform from a soft rubbery state to a hard glassy state. Properties that inform this transition, including the glass transition temperature, *T*_g_, and segmental relaxation times are sought after for polymers. Studies over the past three decades have shown that the *T*_g_ of polymer films often demonstrate the confinement effect, namely, film thickness (*h*) dependences that emerge below ≈100 to 1000 nm (*1*–*9*). The free surface is known to play an essential role as the nearby polymer mobility is much faster than that in the bulk (*10*–*18*). However, a fundamental understanding of this surface effect remains elusive (*4*–*6*, *8*). One obstacle is that the length scale, *h*_t_, by which the fast mobility prevails is still controversial.

By studying the embedding of nanoparticles into the surface of polystyrene (PS), Ilton *et al.* (*15*) found that the PS surface was rubbery even at a temperature, *T*, 80 K below the *T*_g_ of bulk PS, *T*_g,bulk_ (≈373 K). Moreover, the maximum embedding depth, which manifests *h*_t_, was ≈5 nm. By incorporating a low concentration of molecular reporters of segmental dynamics [which are the molecular dynamics (MD) that govern the glass transition in polymer] to free-standing PS films, Paeng *et al.* (*19*) deduced that enhanced segmental dynamics exist within several nanometers of the free surface. Numerous MD simulations also found that *h*_t_ is several nanometers (*16*, *17*, *20*–*22*). However, some experiments suggested that *h*_t_ is micrometers: Pfromm and Koros (*7*) found that the gas permeability of polymer membranes with *h* < ~1 μm after hours-long aging at 293 to 323 K were suppressed relative to their identically aged but thicker counterparts, indicating that the segmental dynamics of the submicrometer membranes were faster. Boucher *et al.* (*23*) identified *T*_g_ to be the temperature whereat the enthalpies of 2-hour aged and unaged PS films began to depart from each other and found that the *T*_g_ deviated from *T*_g,bulk_ starting from *h* ≈ 6 μm. Both findings indicate that *h*_t_ ~ μm.

Dynamic mechanical analysis (DMA) is one of the most common techniques used to study the dynamics of polymers (*24*). However, their application in polymer nanofilms is scarce. Here, by using DMA, we perform a comprehensive relaxation study on free-standing PS and PS–polydimethylsiloxane (PDMS) bilayer films with PS thickness, *h* = 5 nm to 185 μm. A previous experiment showed that the *h* dependences of the *T*_g_ of the two kinds of films are the same [see the supporting information of (*25*)]. As we will see below, our dynamic mechanical data further show that PS and PS-PDMS exhibit the same relaxation properties. These findings substantiate the idea that the PDMS and free surfaces modify the PS dynamics similarly. Our main result reveals that the mobile surface region is dynamically inhomogeneous: In the 1- to 10-nm outmost region, the polymer mobility is faster and caused by surface-enhanced segmental motions. In the ~1-μm region beneath, the enhanced molecular motions are uncorrelated with the segmental ones and attributable to an unknown long-ranged dynamic mode. Therefore, studies probing different kinds of enhanced molecular motions may find different *h*_t_’s.

## RESULTS

**Fig. 1.**
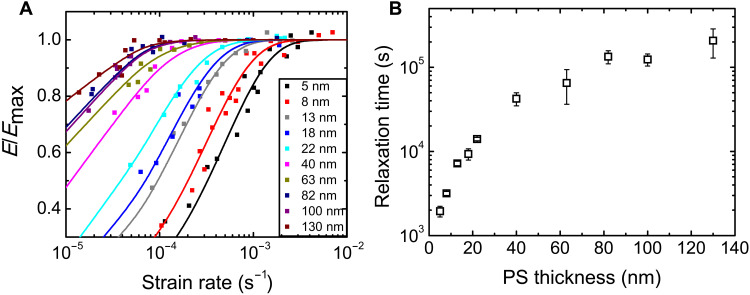
Relaxation data of the *h* ≤ 130-nm-thin PS films supported by PDMS. (**A**) Normalized elastic moduli of PS-PDMS films with various thicknesses as a function of strain rate. The solid lines are the best fits to eq. S1 as described in Materials and Methods. (**B**) A plot of the relaxation times deduced from the data of (A) as a function of PS thickness.

We first discuss measurements of the PS-PDMS nano films with *h* = 5 to 130 nm. The elastic modulus, *E*, was determined from slopes of the stress-strain curves acquired at various strain rates $\overset{\cdot }{\gamma }$ at *T* = 295 K. The result is shown in Fig. 1A as *E*/*E*_max_ versus $\overset{\cdot }{\gamma }$, where *E*_max_ is the maximum value of *E* and given in table S1. As seen, *E*/*E*_max_ increases with $\overset{\cdot }{\gamma }$ initially and then levels off to one after reaching a characteristic $\overset{\cdot }{\gamma }$ that decreases with *h*. On fitting the data to eq. S1, we deduced the relaxation time, τ, of the films and plotted them versus *h* in Fig. 1B. As seen, τ increases with *h* initially and then becomes a constant of ~2 × 10^5^ s when *h* exceeds ≈80 nm. Such an *h*-dependence is consistent with there being a gradient of enhanced mobility in the surface nanoregion, as is broadly observed in MD simulations (*16*, *17*, *20*–*22*). However, unlike MD simulations, where various dynamical properties, such as *T*_g_ and segmental relaxation time, become equal to the bulk value beyond *h* ≈ 100 nm (*16*, *17*), the plateau value of τ in Fig. 1B is orders of magnitude smaller than the bulk relaxation time (*26*).

**Fig. 2.**
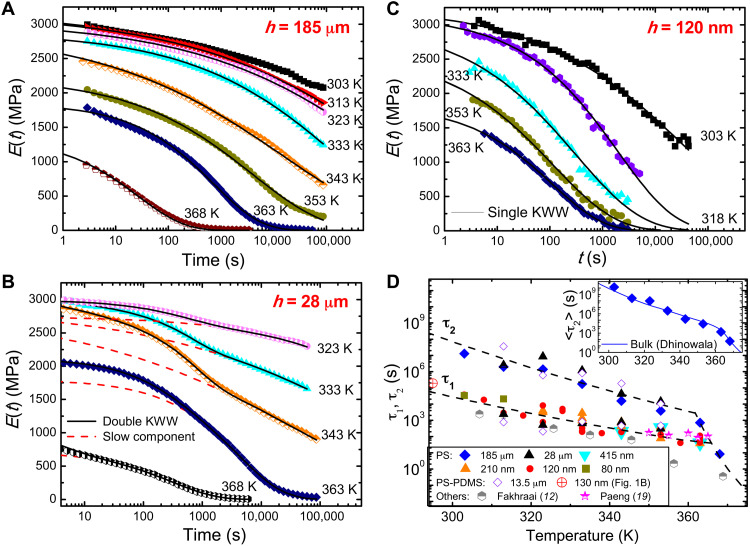
Representative relaxation moduli, *E*(*t*), and relaxation times of the studied films. (**A** to **C**) *E*(*t*) of bulk-like films (*h* = 185 μm), intermediate-thickness films (*h* = 28 μm), and thin films (*h* = 120 nm), respectively, of free-standing PS at different temperatures, *T*, marked on the plots. (**D**) Plots of the relaxation times, τ_1_ and τ_2_ of the fast and slow mode, respectively, of free-standing PS (80 nm ≤ *h* ≤ 185 μm, solid symbols) and PS-PDMS [*h* = (13.5 ± 1.5) μm, open diamonds] as a function of *T*. The relaxation time of the 130-nm PS-PDMS, displayed in Fig. 1B, is shown by the red ringed cross. The surface relaxation times of Fakhraai and Forrest (*12*) (half shaded hexagons) and Paeng *et al.* (*19*) (half shaded stars) are also shown for comparison. The dashed lines are guides to the eye only. The inset depicts the average of τ_2_ plotted as a function of *T* with the same horizontal scale as the main panel. The published data of Dhinojwala *et al.* (*26*) for <τ_bulk_ > are shown by the solid line.

To investigate how the film recovers the bulk dynamics with increasing *h*, we measured the relaxation modulus, *E*(*t*), as a function of time, *t*, from free-standing PS films with *h* = 80 nm to a bulk thickness of 185 μm. The result, obtained for *h* = 185 μm, 28 μm, and 120 nm at various *T* are displayed in Fig. 2 (A to C), respectively. Generally, *E*(*t*) decays faster with higher *T*. At a given *T*, the time scale and shape of *E*(*t*) vary noticeably with *h*. Focusing on the data of 333 K (cyan triangles), the relaxation time scale of the 120-nm films is shorter than that of the 185-μm ones. For the 28-μm films, the data exhibit a step-like feature, suggesting that two relaxation modes coexist. All our *E*(*t*) data can be fitted to the double Kohlrausch-Williams-Watts (KWW) function

$$E(t)=E_{1}\text{exp}[-{\left(\frac{t}{\tau_{1}}\right)}^{\beta_{1}}]+E_{2}\text{exp}[-{\left(\frac{t}{\tau_{2}}\right)}^{\beta_{2}}]$$ (1)

where, without loss of generality, *i* = 1 (2) denotes the fast (slow) mode and *E*_i_ / (*E*_1_ + *E*_2_), τ_i_ and β_i_ are the relaxation strength, relaxation time, and stretching exponent of mode *i*, respectively. Given this convention, *E*_1_ = 0 for *h* = 128 μm and *E*_2_ = 0 for *h* = 120 nm, corresponding to single KWW relaxations.

The solid symbols in Fig. 2D display τ_1_ and τ_2_ versus *T* of free-standing PS films with 80 nm ≤ *h* ≤ 185 μm. As expected, only the 28-μm data display two τ_i_’s. Data of the 185-μm films overlap with the (slow) τ_2_ of the 28-μm films, while those of the *h* ≤ 415-nm films overlap with the (fast) τ_1_ of the 28-μm films. Dhinojwala *et al.* (*26*) have measured the average segmental relaxation times of bulk PS, <τ_bulk_ > (≡ τ_bulk_Γ(1/β_bulk_)/β_bulk_, where Γ is the gamma function). We evaluated <τ_2_> of our 185-μm films by using the data of τ_2_ and β_2_ (shown in fig. S2A; data of β_1_ are also shown for comparison) and plotted the result in the inset of Fig. 2D. The excellent agreement between our data (diamonds) and those of Dhinojwala *et al.* (*26*) (solid line) indicates that our slow mode originates from bulk segmental dynamics.

To connect the data of Fig. 1 with those of Fig. 2, the relaxation time of the 130-nm PS-PDMS films displayed in Fig. 1B was added to Fig. 2D (as the red ringed cross). This data point agrees with the τ_1_ data of the free-standing PS films (solid symbols). We have also measured *E*(*t*) of PS-PDMS bilayers with *h* = (13.5 ± 1.5) μm and therefrom deduced their τ_1_ and τ_2_. The data, denoted by open diamonds in Fig. 2D, evidently agree with the respective ones of the free-standing films. These results demonstrate that our free-standing PS and PS-PDMS films display the same dynamics. We attribute this finding to the fact that our PS-PDMS were not thermally annealed above the *T*_g_ of PS after construction, which, according to Baglay and Roth (*27*), was necessary for the polymer-polymer interface to attain equilibrium and produce a long-range *T*_g_ profile across the interface.

**Fig. 3.**
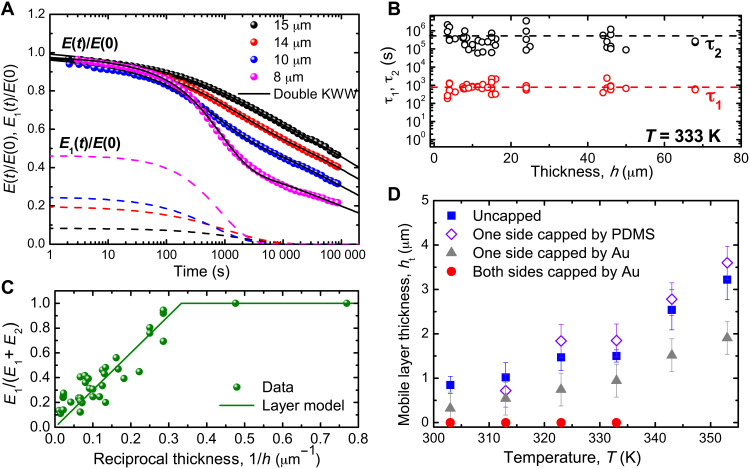
Data demonstrating the existence of two independent relaxation modes in microscale films and their properties. (**A**) Normalized relaxation moduli of free-standing PS films with various microscale thicknesses as a function of time, *t* (symbols). The solid lines are the best fits to the double KWW function. The dashed lines are *E*_1_(*t*)/*E*(0) of the fast mode inferred from the fits. (**B**) Plots of τ_1_ and τ_2_ versus *h* for *T* = 333 K. (**C**) Plot of the fraction of the fast component in the films, *E*_1_/(*E*_1_ + *E*_2_), as a function of reciprocal film thickness, 1/*h*. (**D**) Effective mobile surface layer thickness, *h*_t_, versus temperature *T* measured from pristine free-standing PS films (solid squares) and PS films capped one side by PDMS (open diamonds) or by gold (triangles), and PS films capped both sides by gold (circles). The data of the uncapped films at 333 K were obtained by fitting the data of (C) to the layer model as discussed in the text. For the other uncapped films, the used film thicknesses were such that ~0.3 ≤ *E*_1_/(*E*_1_ + *E*_2_) ≤ ~0.5. For the one-side and two-side capped films, *h* = (13.5 ± 1.5) μm. When there was more than one measurement, the SD was used for the error bar. When there was only one measurement, the error was taken to be 0.03 *h*, where 0.03 is half the average SD found of *E*_1_/(*E*_1_ + *E*_2_) in (C).

Figure 3A depicts the normalized relaxation modulus, *E*(*t*)/*E*(0) (solid circles) as a function of time, *t*, at 333 K, and its portion due to the fast relaxation mode, *E*_1_(*t*)/*E*(0) (dashed lines), determined by fitting the data of *E*(*t*)/*E*(0) to Eq. 1. While the value of *E*_1_(*t*)/*E*(0) decreases clearly with *h*, its relaxation time appears unchanged. Figure 3B confirms that τ_1_ and τ_2_ are constant with *h*, revealing that the fast mode is uncorrelated with the slow mode (or the bulk segmental dynamic mode). Next, we examine *E*_1_(0)/*E*(0) (≡ *E*_1_/(*E*_1_ + *E*_2_)), which signifies the fraction of the fast component in the films. According to the layer model (*10*, *13*), enhanced dynamics exists in the near-surface region and bulk dynamics in the inner region. Provided that *h*_t_ is the thickness of the mobile surface region and constant, the layer model predicts that *E*_1_/(*E*_1_ + *E*_2_) = 2*h*_t_/*h* for *h* ≥ 2*h*_t_ but is 1 for *h* < 2*h*_t_. A plot of *E*_1_/(*E*_1_ + *E*_2_) versus 1/*h* is displayed in Fig. 3C. As one can see, our data agree with the layer model, with *h*_t_ being 1.5 μm.

We have measured *h*_t_ at other temperatures, *T*. The result is plotted versus *T* in Fig. 3D (blue squares). As one can see, *h*_t_ increases with *T*, consistent with the trend found in previous measurements of the thickness of the mobile surface layer (*15*, *19*) [it should be mentioned that the actual *T* dependence of *h*_t_ can be gentler than is shown in Fig. 3D because of limitation of the measurement duration relative to τ_2_ as discussed on pages 3 and 4 of the Supplementary Materials (SM)]. To examine whether the fast mode originates from the free surface, we capped one and both surfaces of the films, respectively, by gold because metal-capping has been found to suppress the free surface effect (*28*). We found that *h*_t_ became half that of the uncapped films for the one-side capped films and zero for the two-side capped films, demonstrating that the fast (τ_1_) mode of our films originates from the near-surface region. In contrast, *h*_t_ of the PS-PDMS bilayers (open diamonds in Fig. 3D) is the same as that of the uncapped films (solid squares). This further demonstrates that the free surface and PDMS surface modify the PS dynamics equally.

**Fig. 4.**
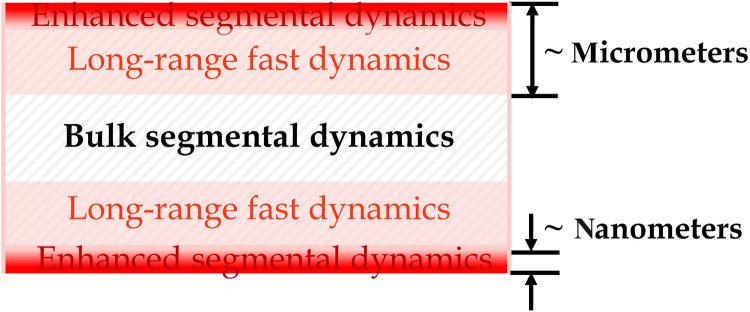
Schematic illustrating the dynamic heterogeneity of the films and the prevailing dynamics in different regions. The central white region denotes the bulk-like region. The red and pink regions denote the (faster) nano–outer layer and (slower) micro-sublayer, respectively, of the mobile surface layer. The types of dynamics prevailing in different regions are also marked: Enhanced segmental dynamics prevails in the nano–outer layer; long-range fast dynamics prevails in the micro-sublayer; bulk segmental dynamics (marked by a hatching pattern) prevails in both the bulk-like region and micro-sublayer of the mobile surface layer.

Notably, the values of *h*_t_ that we found are ~1000 times those found in MD simulations (*16*, *17*, *20*–*22*) and some experiments (*12*, *13*, *19*). To gain some insight, we have added to Fig. 2D the near-surface relaxation time data of Fakhraai and Forrest (*12*) (τ_Fakhraai_, half-filled hexagons)—deduced from spontaneous refilling of nano-depressions on the surface of PS—and those of Paeng *et al.* (*19*) (τ_Paeng_, half-filled stars)—deduced from reporters of segmental dynamics added to the films. τ_1_ ≈ τ_Fakhraai_ from 310 to 350 K. The smaller τ_Fakhraai_ found at *T* > 350 K is attributable to the onset of accelerated growth in the thickness of the mobile surface layer with *T* near 350 K (*15*) whereupon the refilling dynamics sped up. The fact that τ_1_ ≈ τ_Paeng_ for *T* ≥ 350 K before the onset of global glass transition (Fig. 2D) supports this interpretation. The fact that τ_1_ overlaps with τ_Paeng_ and τ_Fakhraai_ over a broad range of *T* reveals that the fast (τ_1_) mode originates from the near-surface nanoregion wherefrom Paeng *et al.* (*19*) and Fakhraai and Forrest (*12*) accessed the fast dynamics. On the other hand, the *h*_t_ values of our films (Fig. 3D) show that the fast mode of the microscale films is of micrometer range. Moreover, it is independent of the slow, local (bulk-like) segmental motions (Fig. 3B), unlike the fast mode of the *h* ≤ 130-nm films (Fig. 1) that is relatable to the local (faster) segmental motions probed by Paeng *et al.* (*19*) and Fakhraai and Forrest (*12*). These observations suggest that there are two types of fast modes: One is localized in the nanoscale near-surface region and caused by the enhanced segmental dynamics therein; the other one is long-range, found in the microscale films. Figure 4 summarizes the dynamic heterogeneity in the films as revealed by the current findings and the prevailing types of dynamics in each of the dynamically distinct regions.

The dynamic characteristics like those found of the long-range fast mode have not been reported before. It is also unclear how this mode may have originated from the near-surface nanoregion and be long-range. A recent atomistic MD simulation study of Zhou and Milner (*17*) of free-standing PS films illuminates a possibility. Besides the usual surface-enhanced segmental dynamics, these authors also observed longer-range collective motions attributable to the spontaneously excited surface shear modes (fig. S3). We have estimated the propagation distance, *l*, of these modes and found that *l* can be ~1 μm (see the calculation on page 3 of the SM). Because the excitation volume of the mode is ~1000 nm^3^, which is much larger than the cooperativity size of bulk polymer at the glass transition [~1 nm^3^; (*29*)], the surface shear mode and bulk segmental mode must not be coupled. This expectation is fulfilled by the independence between the long-range fast mode (τ_1_) and bulk segmental mode (τ_2_) shown by the data of Fig. 3B.

As mentioned in the introduction, Boucher *et al.* (*23*) found that the *T*_g_ of polymer films began to deviate from *T*_g,bulk_ at a film thickness of several micrometers. By assuming that excess free volume holes existed in the films and escaped during measurement by diffusion alongside a second mechanism that has an inherent length scale [or so-called internal length scale (*23*, *30*)] of micrometers (*23*), these authors were able to model their data. Models using similar assumptions can likewise fit the gas permeability data of polymer membranes exhibiting confinement effect, wherefrom the internal length scale was also found to be micrometers (*30*, *31*). However, the origin of the second mechanism and thereby the internal length scale remains elusive. Our long-range fast mode, with a microscale propagation distance, may provide a natural explanation to the internal length scale. Revelation of a micrometer internal length scale in (*7*, *23*, *30*) and the like (*31*) would show that thickness of the mobile surface layer is microscale. On the other hand, experiments designed to probe nanoscale relaxations (*12*) or segmental motions (*17*, *19*) would detect the surface-enhanced segmental motion only and reveal a nanoscale mobile surface layer (*12*, *17*, *19*). These deliberations may explain the vastly different thickness values of the mobile surface layer found in the literature.

The micrometer-ranged fast mode can also explain another conundrum of the confinement effect, namely, the dynamic decoupling of polymer films (*4*, *5*, *23*, *32*)—an enigmatic phenomenon in which polymer films, including those that reveal large departures of the *T*_g_ from *T*_g,bulk_, exhibit segmental relaxation times like those of the bulk polymer (*4*, *5*, *23*, *32*). For nanoscale films, the dynamic decoupling was explained by Schweizer and Simmons (*8*) on the basis of the existence of a gradient of activation energy barrier in the nanoscale near-surface region. For microscale films, a fundamental explanation is still lacking. The micrometer-ranged fast mode found here, with no coupling with the segmental dynamics, allows for a natural explanation to the dynamic decoupling in the microscale films.

## DISCUSSION

The relaxation time and relaxation strength of the surface-enhanced relaxation mode of PS films with thicknesses of 5 nm to 185 μm were measured. It was found that the physical origin of this surface-induced relaxation enhancement effect varies with distance from the surface: Within the nanoscale outer region, the enhancement effect is caused by the local segmental motions, which are themselves enhanced; beneath this region, where the segmental motions cease to be enhanced, the enhancement effect is dominated by an exotic long-range fast mode uncorrelated with the bulk segmental motions. These findings can account for why different studies have found different apparent thicknesses of the mobile surface layer and the phenomenology of the dynamic decoupling of polymer films. Future works should elucidate the fundamental origin of the long-range fast mode, for which the surface shear mode is a worthy candidate.

## MATERIALS AND METHODS

### Method to make free-standing PS and PS-PDMS films

PS, with a weight-average molecular weight, *M*_w_, of 1119 kg/mol and polydispersity index of 1.06, was purchased from Scientific Polymer Products (Ontario, NY). The as-purchased polymer was dissolved in toluene and then filtered through a 0.2-μm pore-sized polytetrafluoroethylene membrane filter (Fisher Scientific Co.).

The films studied in this work include free-standing PS with thickness *h* between 80 nm and 185 μm and PS supported by a ~20-μm-thick PDMS (PS-PDMS) with *h* between 5 nm and 15 μm. As demonstrated in the main text, these two kinds of films exhibit the same dynamics.

**Fig. 5.**
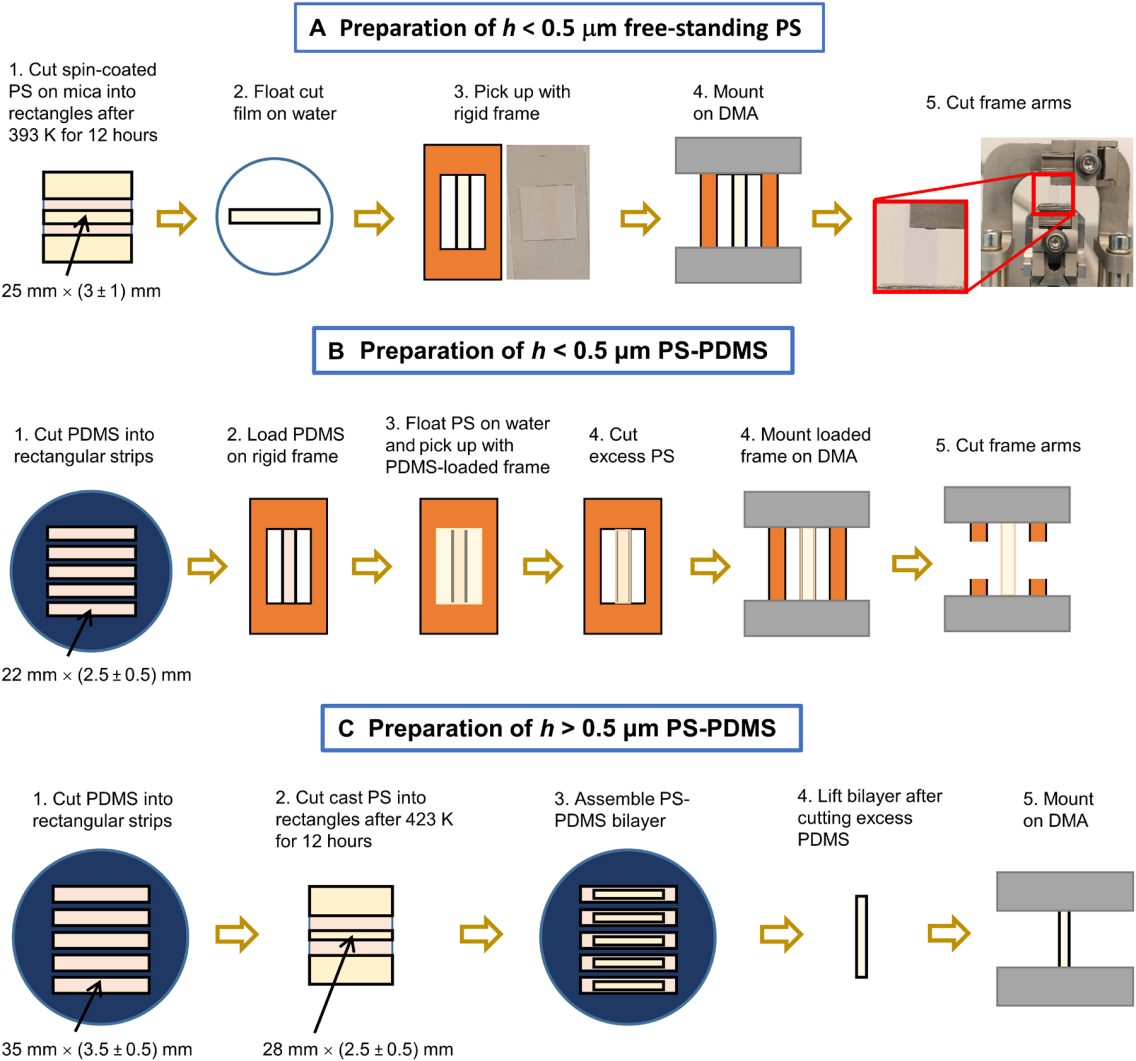
Schematics showing how the free-standing PS with *h* < 0.5 μm and PS-PDMS bilayers of all *h* were prepared. (**A**) Procedure for preparing free-standing PS with *h* < 0.5 μm, (**B**) PS-PDMS with *h* < 0.5 μm, and (**C**) PS-PDMS with *h* > 0.5 μm, respectively.

If PS films with *h* < 0.5 μm were to be made, then the films were coated on freshly cleaved mica by spin coating and then annealed in a vacuum oven (evacuated by an oil-free pump) at 393 K for 12 hours. Afterward, the films were allowed to cool inside the vacuum oven to room temperature. The film thickness was determined by ellipsometry (LSE Stokes Ellipsometer by Gaertner Scientific Corp.) and varied by adjusting the solution concentration between 0.5 and 4%, while fixing the spinning speed at 3500 rpm. Afterward, the films were cut into ~25 mm by (3 ± 1) mm rectangles by a razor blade before floating onto a rigid supporting frame (which was hollow for making free-standing PS films or preloaded with a PDMS layer for making PS-PDMS bilayers) by a water transfer technique (*25*). Thereafter, the films were dried in a vacuum oven for ~10 hours at room temperature. The schematics shown in Fig. 5 (A and B) illustrate how these films were prepared.

To make the PDMS supporting layer, the elastomer base and curing agent from two-part PDMS kits (Sylgard 184, Dow Corning) were mixed in a 10:1 mass ratio. After degassing under vacuum for 0.5 hours, the mixture was spin-coated onto a PS petri dish at 1000 rpm for 10 s, followed by 3000 rpm for 60 s. Afterward, the films were cured at 343 K for 1.5 hours. This makes PDMS layers with a thickness of ~20 μm and elastic modulus, *E*_PDMS_, of 0.9 to 1.6 MPa.

For the PS films with *h* above 0.5 μm, the filtered polymer solution was cast into a Teflon mold with *h* controlled by the mass of the PS in the cast solution. The Teflon mold was then placed inside an enclosure and allowed to evaporate slowly under ambient conditions for 24 hours. Thereafter, the mold was annealed at 423 K in a vacuum oven for 24 hours and cooled inside the oven to room temperature afterward. The cooled film was removed from the Teflon mold and cut into ~22 mm by (2.5 ± 0.5) mm strips for use as single-layer films. To make PS-PDMS bilayers (i.e., one-side PDMS-capped films), individual PS strips were carefully laid onto ~35 mm by (3.5 ± 0.5) mm by 20 μm PDMS strips. Excess PDMS was removed by a razor blade (Fig. 5C).

One-side and two-side gold-capped PS films were obtained by sputtering ~50 to 150 nm of gold on one side and both sides, respectively, of PS films by using a Desk II Denton sputtering system under a 75-millitorr vacuum. The growth rate was controlled to 0.5 Å/s, as the gold thickness was monitored in situ by a quartz crystal sensor. The final thickness of the gold coating was determined by step profiling with a Seiko Instruments SPA-300 HV atomic force microscope.

Pye and Roth (*31*) have found that when supported polymer films were quenched from a temperature above the *T*_g_ to a lower one below the *T*_g_, stresses developed in the films because of thermal-expansion mismatch between the polymer and substrate. The presence of these stresses quickens aging in the films. In this experiment, our PS films with *h* < 0.5 μm and *h* > 0.5 μm were supported by different substrates—namely, mica and Teflon, respectively—when they were cooled from the annealing temperature to room temperature. Because the stresses developed in the films depend only on the final temperature (*31*), which was the same for all our films, the stresses in the (*h* < 0.5 μm) mica-supported films should be bigger than those in the (*h* > 0.5 μm) Teflon-supported films. However, no noticeable change in the relaxation time of the fast mode is noticeable across *h* = 0.5 μm (Fig. 2D). This observation demonstrates that the thermal expansion mismatch-induced stresses must be insignificant in this experiment. Two factors may contribute to this finding. First, our films were cooled slowly to room temperature, whereby some relaxations might occur. Second, the PS films were lifted off from the substrate after cooling to form free-standing PS or PS-PDMS films. This step may alleviate some stresses in the films.

### Relaxation modulus measurements

Measurements of relaxation modulus, *E*(*t*), were carried out in a DMA Q800 by TA Instruments. The films (together with the supporting frame if equipped) were clamped onto the DMA to leave a gap of 7 to 15 mm in length. Thereafter, the vertical arms of the clamped frame, if present, were cut. Before measurement, the sample temperature was brought to the desired value and allowed to equilibrate for 10 min. Then, a small strain, γ, of ~0.5% was applied to the film. The ensuing evolution of the measured stress, σ(*t*) [which allows *E*(*t*) = σ(*t*)/γ to be determined], was recorded by the DMA.

### Strain rate–dependent elastic modulus measurements

Measurements of elastic modulus (*E*) versus strain rate ($\overset{\cdot }{\gamma }$) were performed on PS-PDMS bilayers with *h* = 5 to 130 nm. After mounting the films (together with their supporting frames) on the DMA with a gap distance of 10 to 15 mm, the vertical arms of the supporting frame were cut. Then, the *E* versus $\overset{\cdot }{\gamma }$ dependence was determined by taking stress-strain curves of the PS-PDMS bilayers at various $\overset{\cdot }{\gamma }$. The elastic modulus of the bilayer, *E*_bilayer_ was determined from the slope of the stress-strain curve. Because the elastic modulus of the PDMS layer, *E*_PDMS_, also contributes to *E*_bilayer_, we use the rule of mixture to determine the elastic modulus, *E*, of the PS layer (*25*)

$$E=\frac{1}{h}[(h+h_{\text{PDMS}})\cdot E_{\text{Bilayer}}-h_{\text{PDMS}}\cdot E_{\text{PDMS}}]$$ (2)

where *h*_PDMS_ = 18 to 25 μm is the thickness of the PDMS layer and determined by using a surface profiler (Tencor D300, KLA) and *E*_PDMS_ is the elastic modulus of the PDMS layer and determined from slope of the stress-strain curves of the PDMS layer taken before the layer was used to form the bilayer.

### Methods used to fit the data

The data of *E* versus $\overset{\cdot }{\gamma }$ in Fig. 1A were fit to eq. S1 by varying the parameters *E*_max_, τ, and β under the constraint that β was smaller than or equal to that of the next thicker film. Imposition of this constraint is justified by the observation that the dynamics of polymer nanofilms exhibits the Arrhenius temperature dependence, whereas that of the thicker polymer films (*h* ≥ ~100 nm) exhibits the Vogel-Fulcher-Tammann temperature dependence, which indicates that thicker films have greater dynamic heterogeneity (*13*).

The data of *E* versus *t* were fit to Eq. 1 with fit parameters *E*_1_, τ_1_, β_1_, *E*_2_, τ_2_, and β_2_. We found that imposition of constraints like the one above frequently led to inability of the fits to converge. Therefore, for the *E* versus *t* data, we applied no constraint to our fitting procedure.

Unless otherwise stated, we used the SE of a fit parameter reported by our fitting program for the error bar. However, as can be seen from Figs. 2D and 3 (B and C) and fig. S2, the scattering of data is frequently bigger than the SE. This shows that our experimental uncertainty is dominated by reproducibility of the measurement.

When the maximum measurement time (*t*_max_) is shorter than the relaxation time, the KWW fit parameters may deviate from the actual values—which are obtained when *t*_max_ is sufficiently longer than the relaxation time. To assess the magnitude of this effect, we have fit the data of the 185-μm PS film at 353 K (Fig. 2A) after truncating them at various *t*_max_’s between 0.01τ_2_ and 21τ_2_. The hence obtained fit values of τ_2_, *E*_max_, and β are displayed in fig. S4 (A to C) as a function of *t*_max_/τ_2_. The result shows that the effect of changing *t*_max_/τ_2_ on τ_2_ is small. However, for β and *E*_max_ (and thereby *h*_t_), the more *t*_max_/τ_2_ is decreased below one, the more β and *h*_t_ are underestimated. Consequently, the temperature dependences of β and *h*_t_ may appear steeper than they are at low temperatures. More detailed discussions can be found on pages 3 and 4 of the SM.
