# Supplementary material for: Microscale mobile surface double layer in a glassy polymer
Source: Sci Adv. 2022 Nov 9;8(45):eabq5295. doi: 10.1126/sciadv.abq5295 (PMC9645724; doi:10.1126/sciadv.abq5295)
Supplement: Supplementary file 1 — Supplementary Text Figs. S1 to S4 Table S1 [file sciadv.abq5295_sm.pdf]

Science Advances
AAAS

Supplementary Materials for

Microscale mobile surface double layer in a glassy polymer

Hailin Yuan *et al.*

Corresponding author: Ophelia K. C. Tsui, okctsui@ust.hk

Sci. Adv. **8**, eabq5295 (2022)
DOI: 10.1126/sciadv.abq5295

This PDF file includes:

Supplementary Text
Figs. S1 to S4
Table S1

Supplementary Text

A. E_{max} of the PS-PDMS films in Fig. 1A

Table S1 shows the values of E_{max} of the PS-PDMS films in Fig. 1A. The minor reduction of E_{max} from ~3.18 GPa at h = 130 nm to 2.7 GPa at h = 5 nm is consistent with the argument given in Ref. (*25*) that $E \sim \rho$, where ρ is the density of polymer and it had been found that ρ tapered from the bulk value to zero near the free surface within ≈ 1 nm (*17*).

B. Model equation for the E vs $\dot{\gamma}$ data of PS-PDMS and relaxation behavior of the PDMS supporting materials

The following equation was used to fit the E vs $\dot{\gamma}$ data of PS-PDMS in Fig. 1A (*25*):

$$E(\dot{\gamma}) = E_{\max}\left\{1-\exp\left[-\left(\dot{\gamma}\tau\right)^{\beta}\right]\right\} \qquad \text{(S1)}$$

where $\dot{\gamma}$ is strain rate, $E_{max} \equiv \lim_{\dot{\gamma}\to\infty} E(\dot{\gamma})$ is the instantaneous elastic modulus and β is the stretching exponent. The fits, denoted by solid lines in Fig. 1A, provide a good description of the data.

Figure S1 displays the analogous relaxation data of the PDMS supporting material. As one sees, the data exhibits no noticeable dependence on $\dot{\gamma}$. This shows that the varying $E(\dot{\gamma})$ dependences of the PS-PDMS films seen in Fig. 1A must originate from the PS layer.

C. Stretching exponents of the PS films exhibiting the single-KWW relaxation

The values of β_1 and β_2, deduced by fitting the single-KWW function (i.e., Eq. 1 with either E_1 or E_2 set equal to zero) to the h < 0.5 μm and h = 185 μm free-standing PS films, respectively, are plotted as a function of temperature in Fig. S2. As one can see, both β_1 and β_2 increase with T, consistent with the common observation that the dynamic heterogeneity of glass formers like PS diminishes with temperature below the T_g. However, it should be mentioned that the actual trend of β_2 versus T can be gentler than is shown by Fig. S2 due to limitation of the measurement time as discussed on pp. 3–4.

We further observe that β_1 is on average bigger than β_2. This is in keeping with the general finding that the fast mode corresponds to dynamics in the near-surface region, where the T_g is lower than the bulk T_g. We did not include data of the films exhibiting double-KWW relaxations

as we have found that the fit values of β_1 and β_2 of these films were too scattered to allow meaningful insights to be drawn.

D. Shear excitations near the polymer free surface

Figure S3 illustrates the shear excitation mode discussed in the main text. Here, we estimate the maximum lateral displacement, ξ, of the mode when the propagation length, l, is ~h_t or ~1 μm. Because the shear mode occurs spontaneously, its energy, U, is ~k_BT, which gives:

$$U \approx \frac{1}{2} G \left(\frac{\xi}{l}\right)^2 \left(\xi^2 l\right) \approx k_B T \ , \tag{S2}$$

where G is the shear modulus of the polymer and k_B is the Boltzmann constant. Assuming that the Poisson's ratio is 0.5 and $G \sim E/3$. Equation S2 can be rewritten as:

$$\xi \approx \left(\frac{6k_B Tl}{E}\right)^{\frac{1}{4}} . \tag{S3}$$

By substituting E = 3 GPa (*25*), T = 300 K and l ~ 1 μm, one finds that ξ ~ 1 nm, which is in good consistency with the simulation result of Zhou and Milner (*17*) and expectations because the thickness of the surface nanolayer is ~1 nm and so the maximum molecular displacement therein should also be ~1 nm (along any directions due to isotropy of the thin film material).

E. Effect of the measurement time relative to the relaxation time on the KWW model fit

In the $E(t)$ data taken below ~330 K, the maximum measurement time (t_{max}) was shorter than the slow relaxation time, τ_2. To address if this may affect the parameters obtained from the KWW fit, we refit the data of 185 μm PS films at 353 K (Fig. 2A) upon truncating the data at different t_{max} values of $5\tau_2$, $2\tau_2$, τ_2, $0.5\tau_2$, $0.2\tau_2$, $0.05\tau_2$, $0.02\tau_2$, and $0.01\tau_2$, where 4170 s is the value of τ_2 obtained when the original data, with $t_{max} = 20.7\tau_2$, was fit to the single KWW equation (i.e., Eq. 1 with $E_1 = 0$). We have opted to examine a case exhibiting single KWW relaxations only because errors are large for cases exhibiting double KWW relaxations (six variable parameters), whereby the uncertainties may obscure our goal to investigate impacts of the relative measurement time (t_{max}/τ_2) on the fit parameters.

Figures S4A–C (black symbols) display the result we obtained for τ_2, E_{max} and β plotted as a function of t_{max}/τ_2. As one can see, with a decrease of t_{max}/τ_2 from 21, τ_2 remains constant until

t_{max}/τ_2 reaches 0.05 below which τ_2 is suppressed. However, the size of the suppression remains small relative to the scattering of the data of τ_2 (and τ_1) in Fig. 2D. At the same time, the error bar of τ_2 becomes larger than the size of the symbols (Fig. S4A). Because none of our measurements in Figs. 2D show error bars larger than the size of symbols, we think that the relaxation times we measured were not significantly different from the long-t_{max} value even when our t_{max}/τ_2 vales are decreased one.

In comparison, both E_2 and β_2 vary more noticeably when t_{max}/τ_2 is decreased below 1. Specifically, as t_{max}/τ_2 is decreased from 21 to 0.05 (i.e., just before the error bar of τ_2 becomes visibly enlarged), E_2 and β_2 is increased and decreased, respectively, by ~50% from the long-t_{max} values (Figs. S4B and S4C). This shows that the increase of β_2 in Fig. S2 from ~0.2 to ~0.5 as T was increased from ~300 K to ~370 K can be partly caused by the approach of t_{max}/τ_2 to 1 from below. Furthermore, because the slow mode is more susceptible to the effect of t_{max}/τ_2 falling below one than the fast mode is and the data taken at low temperatures are more susceptible to this effect than those taken at higher temperatures are, the ratio of $E_1/(E_1 + E_2)$ and hence the value of h_t can be depressed more at lower temperatures. All these imply that the temperature dependence of h_t we measured (Fig. 3D) can appear steeper than it is. Similarly, the temperature dependence of β_2 (Fig. S2) may also be steeper than the real one.

The afore-discussed effect of data truncation on the KWW fit parameters were attained by discarding data beyond t_{max}. As a result, the truncated data with a smaller t_{max}/τ_2 have fewer data points, which may contribute to the increase in size of the error bars found at small t_{max}/τ_2 (Figs. S4A–C). To remove this factor, we repeated the above analysis by adding data points by interpolation while keeping the total number of data points constant. The result is shown by the red symbols in Figs. S4A–C. As we can see, the size of error bars diminishes with the addition of data as expected. But the effects of decreasing t_{max}/τ_2 on the KWW fit parameters and size of the error bars are qualitatively the same as discussed above.

Fig. S1.

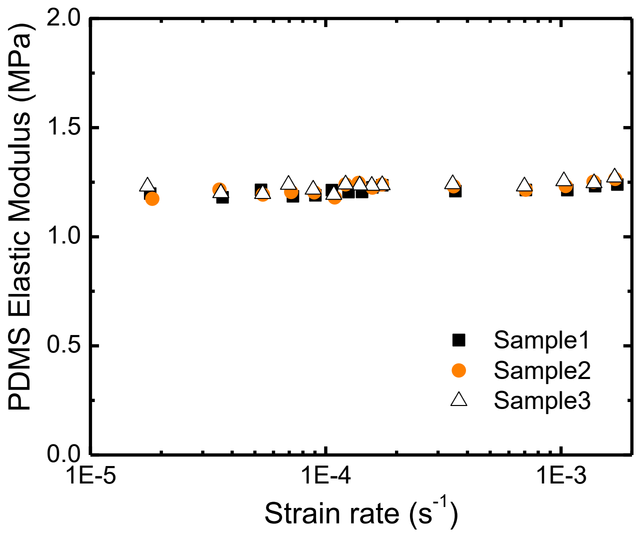

Fig. S1. Relaxation of the PDMS supporting material. Elastic modulus versus strain rate of three PDMS layers with the same thickness of 155 μm and prepared similarly as those used to support the PS films.

Fig. S2.

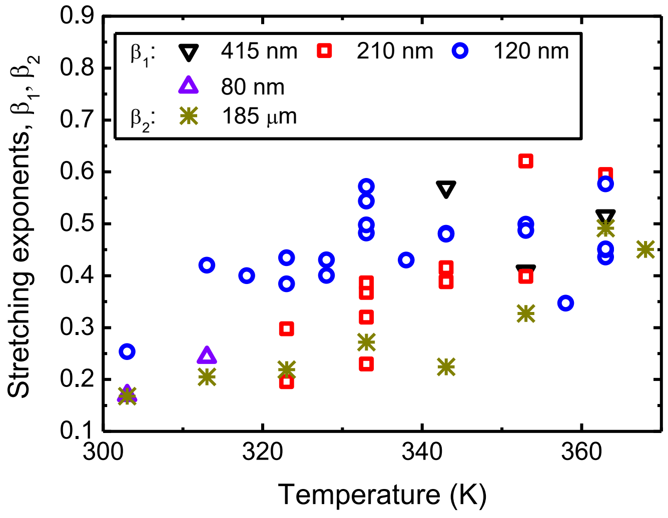

Fig. S2. Stretching exponents, β_1 and β_2, of the single-KWW relaxation of the *h* < 0.5 μm and *h* = 185 μm free-standing PS films, respectively. Here, the data are plotted as a function of temperature.

Fig. S3.

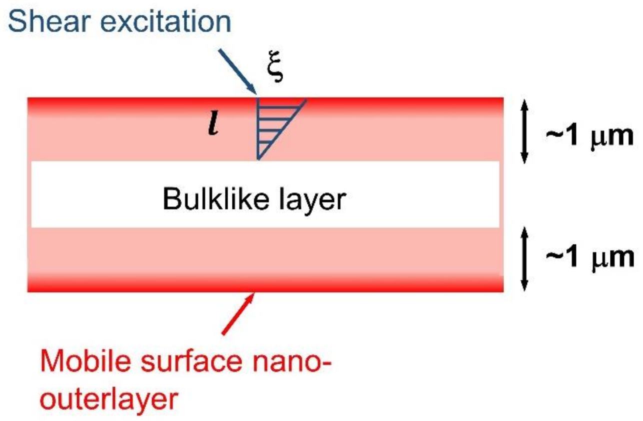

Fig. S3. Illustration of the shear excitation mode discussed in the main text. The main drawing shows the layered dynamic structure of a polymer film possessing two mobile surface layers (red for the faster surface nano-outerlayer and pink for the surface micro-sublayer) and a middle bulklike layer (white). The shear excitation mode has a linear velocity profile as found in MD simulations (*17*). The parameters, ξ and l discussed in the main text are also shown. A calculation based on the requirement that the energy of the mode is $\sim k_B T$ (Eq. S2) supports the value of l to be $\sim h_t$ provided $\xi \sim 1$ nm.

Fig. S4.

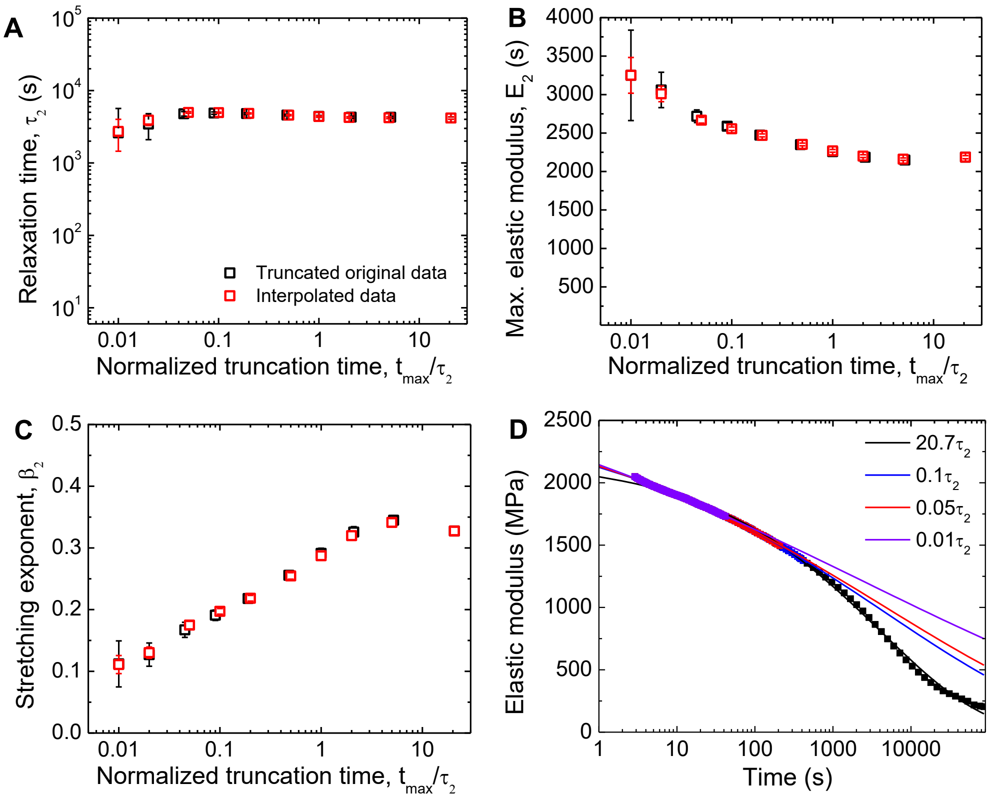

Fig. S4. Effect of truncation time (t_{max}) on the KWW fit. Here, the $E(t)$ data of the 185 μm films at 353 K is used as the model data. The fit results of (**A**) τ_2, (**B**) E_2, and (**C**) β_2 are plotted as a function of normalized truncation time, t_{max}/τ_2. The symbol size is the same as those in Figs. 2D and S2. (**D**) A sample of four fit lines (solid lines) obtained when $t_{max} = 20.7\tau_2$, $0.1\tau_2$, $0.05\tau_2$ and $0.01\tau_2$, respectively. The data with $t_{max} = 20.7\tau_2$ (black symbols) is the original data. The other data were produced by interpolation.

Table S1.

h (nm)	E_{max} (GPa)
5	2.70 ± 0.09
8	3.00 ± 0.07
13	3.02 ± 0.01
18	3.10 ± 0.04
22	3.18 ± 0.07
40	3.17 ± 0.04
63	3.18 ± 0.03
82	3.19 ± 0.01
100	3.18 ± 0.01
130	3.18 ± 0.02

Table S1. The values of E_{max} in Fig. 1A
